# Rosenroot (*Rhodiola*): Potential Applications in Aging-related Diseases

**DOI:** 10.14336/AD.2018.0511

**Published:** 2019-02-01

**Authors:** Wei Zhuang, Lifeng Yue, Xiaofang Dang, Fei Chen, Yuewen Gong, Xiaolan Lin, Yumin Luo

**Affiliations:** ^1^Department of Pharmacy, Xuanwu Hospital of Capital Medical University, Beijing 100053, China; ^2^Dongzhimen Hospital, Beijing University of Chinese Medicine, Beijing 100700, China; ^3^Department of Pharmacy, Hospital of T.C.M.S Shijingshan District, Beijing 100043, China; ^4^College of Pharmacy, University of Manitoba, Winnipeg R3E 0T5, Manitoba, Canada; ^5^Institute of Cerebrovascular Disease Research and Department of Neurology, Xuanwu Hospital of Capital Medical University, Beijing 100053, China

**Keywords:** *Rhodiola rosea*, salidroside, neurodegenerative diseases, cardioprotection

## Abstract

Aging is a progressive accumulation of changes in the body, which increases the susceptibility to diseases such as Alzheimer’s disease, Parkinson’s disease, cerebrovascular disease, diabetes, and cardiovascular disease. Recently, Chinese medicinal herbs have been investigated for their therapeutic efficacy in the treatment of some aging-related diseases. *Rhodiola*, known as ‘Hongjingtian’ in Chinese, has been reported to have anti-aging activity. Here, we provide a comprehensive review about its origin, chemical constituents, and effects on aging-related diseases.

The genus *Rhodiola* in the family Crassulaceae contains herbaceous perennial plants that often occur as fleshy creeping rhizomes. *Rhodiola* species are also commonly known as rosenroot, golden root and orpin rose. Throughout history, they have been considered valuable medicinal plants in China, Europe, and North America.

There are 96 species of *Rhodiola* in the world and most are found in different regions of China. China is considered the main growing area of *Rhodiola* with 73 species, two subspecies and seven varieties. Within China, about 90% of *Rhodiola* can be found in the northwest, southwest and northeast regions such as Tibet, Qinghai, Yunnan, Sichuan and other alpine provinces. In the plant classification system, genera have several subcategories including sections, series and species. According to the “Chinese flora”, the genus *Rhodiola* has eight sections. The medicinal herbs in this genus are primarily found in three sections: Sect. Chamaerhodiola (Fisch. et Mey.) A. Bor., Sect. *Rhodiola*, and Sect. Trifida (Frod.) S. H. Fu. The main medicinal *Rhodiola* species are listed in Table 1. *Rhodiola* usually grows in limestone and granite soils at high altitudes (3500 to 5000 m), although a few species can also be found in alpine grasslands or shrublands at altitudes of about 2000 m.

**Table 1:** Species, geographical distributions, and growing environments of medicinal *Rhodiola*.

Section	Series	Latin Name	Geographical Origin	Growing Environment	Altitude (m)
Sect. Chamaerhodiola (Fisch. et Mey.) A. Bor.	Ser. Dumulosae (Frod.) S.H. Fu	*R. dumulosa* (Franch.) S.H. Fu	Sichuan, Shanxi, Gansu, Ningxia, Qinghai, Shanxi, Hebei, Inner Mongolia	Slopes, rocks	1600-3900
	Ser. Quadrifidae (Frod.) S.H. Fu	*R. quadrifida* (Pall.) Fisch. et Mey.	Tibet, Sichuan, Xinjiang, Gansu, Qinghai	Alpine meadows, schist on mountain slopes, rock crevices on mountain slopes, marshes	3000-5700
		*R. scabrida* (Franch.) S.H. Fu	Sichuan, Yunnan	Grassland on slopes	3200-4700
		*R. subopposita* (Maxim.) Jacobsen	Gansu, Qinghai	Rock crevices on mountain slopes	1600-5000
		*R. atuntsuensis* (Praeg.) S.H. Fu	Yunnan		
	Ser. Fastigiatae (Frod.) S.H. Fu	*R. fastigiata* (Hook. f. et Thoms.) S.H. Fu	Tibet, Sichuan, Gansu, Yunnan	Schist on mountain slopes, slopes, rock crevices	3300-5400
		*R. pamiroalaica* A. Bor.	Xinjiang	-	2000-4200
		*R. himalensis* (D. Don.) S.H. Fu	Sichuan	-	3700-4200
		*R. tangutica* (Maxim.) S.H. Fu	Sichuan, Gansu, Ningxia, Qinghai	Rock crevices on mountain slopes, meadows, around water	2000-4700
Sect. Rhodiola	Ser. Roseae (Praeg.) S.H. Fu	*R. rosea* L.	Xinjiang, Qinghai	Alpine grasslands, under forest, beside ditches	1800-2035
		*R. sachalinensis* A. Bor	Heilongjiang, Jilin	Under hills and trees, under rocks	1700-2300
		*R. crenulata* (Hook. f. et Thomas) H. Ohba	Tibet, Sichuan, Qinghai	Alpine gravel beach, slopes, grasslands, rock crevices	3400-5600
		*R. kirilowii* (Regel) Maxim.	Qinghai, Sichuan, Xinjiang, Shanxi, Gansu	Schist on mountain slopes, under rocks in the forest, meadows, beside ditches	3100-5600
		*R. linearifolia* A. Bor	Xinjiang	-	2000-4200
	Ser. Bupleuroides Frod.) S.H. Fu	*R. bupleuroides* (Wall. ex Hook. f. et Thoms.) S.H. Fu	Tibet, Sichuan, Qinghai	Hillside flow, alluvial plain, subalpine meadow, marshes, grassland	2400-5600
	Ser. Yunnanenses (Frod.) S.H. Fu	*R. yunnanesis* (Franch.) S.H. Fu	Tibet, Sichuan	Rocks under forest, rocks beside ditches	2750-3200
		*R. henryi* (Diels) S.H. Fu	Sichuan, Shanxi, Gansu	Slopes, beside ditches, rocks	1000-3300
Sect. Trifida (Frod.) S.H. Fu	-	*R. sacra* (Prain ex Hamet) S.H. Fu	Tibet, Qinghai	Rock crevices on mountain slopes, grassland on slopes	3500-4700

*Rhodiola* is also known as “oriental god grass” and “plateau ginseng” and has very good value for medicine and healthy living. According to the basic tenets of traditional Chinese medicine, it can boost qi and dissipate blood stasis, unblock the blood vessels, relieve pain, fortify the spleen, treat palpitations, relieve coughing and shortness of breath, reduce fatigue and weakness. *Rhodiola* is also used as an anti-aging herb and for the treatment of aging-related diseases. Current pharmacological investigation reveals that *Rhodiola* has therapeutic value for many diseases such as Alzheimer’s disease (AD), Parkinson’s disease (PD), cerebrovascular disease, diabetes, and cardiovascular disease (CVD). The therapeutic actions and pharmacological functions of *Rhodiola* are listed in Table 2. Although *Rhodiola* is a large genus, only a few species have been investigated. In this review, the pharmacology of five species including *R. rosea, R. crenulata, R. kirilowii, R. imbricata*, and *R. sachalinensis* are discussed.

**Table 2:** Pharmacological functions of medicinal *Rhodiola* on various ailments.

Component or extraction method	Ailment	Pharmacological function	Refs
Salidroside	AD	Upregulates p-GSK-3β and downregulates p-tau	5
		Upregulates PI3K/AKT signaling	6,7
		Weakens the abnormal processing of APP	8
		Induces antioxidant enzymes TRX, HO-1, and PRXI	9
		Prevents caspase 3 activation, increases BAX/BCL-2 ratio, and reverses hippocampal neuronal loss	10
		Protects mitochondria against sodium-azide-induced damage	11
	Depression	Reduces TNF-α and IL-1ß levels	21
		Attenuates levels of IL-6 and TNF-α	22
		Attenuates NE and 5-HT levels in the prefrontal cortex	22
		Regulates BDNF/TRKB signaling pathway	23
	Huntington’s disease	Reduces neuronal death and behavioral dysfunction mediated by polyQ	32
		Regulates AMPK/SIRT1/FOXO1 signaling	33
	CVD	Attenuates H_2_O_2_-induced cell damage by downregulating Ca^2+^ and ROS *via* cAMP-dependent pathway	43
		Promotes mitochondrial biogenesis and functions	44, 45
		Increases the phosphorylation of AKT and ERK1/2; reduces the intracellular levels of ROS and the phosphorylation of JNK and p38 MAPK	47
		Reduces the contents of CK, CK-MB, and LDH; increases GSH-Px and SOD activities; and reduces MDA content in liver tissue	48, 49
		Increases levels of VEGF; upregulates HIF-1α protein expression and induces its translocation	49
		Regulates BCL-2 protein family, reduces the expression of BAX; rescues the balance of pro- and anti-apoptotic proteins	50
		Increases phosphorylation of AKT and reduces activation of caspase 3; markedly increases BCL-2/BAX ratio; preserves mitochondrial transmembrane potential	51
	Diabetes	Reduces diabetes-induced oxidative stress	64
		Inhibits the function and expression of Ca_L_ channels in vascular smooth muscle cells	67
		Inhibits neuroinflammation and P2X7 receptor expression	68
	Hepatic fibrosis	Inhibits lipid peroxidation	73
	Acute liver fibrosis	Antioxidant activity and inhibits the function of HIF-1α	74
	Bladder cancer	Inhibits the mTOR pathway and induces autophagy	79
	Lung cancer	Reduces intracellular ROS generation and phosphor-p38 MAPK expression	80
	Fibrosarcoma	Downregulates the ROS/PKC/ERK1/2 signaling pathway	81
	Colon carcinoma	Inhibits the JAK2/STAT3-dependent pathway	82
	Sarcoma	Reduces tumor-induced angiogenesis	83
	Pulmonary hypertension	Regulates ET-1, NO, VEGF, ACE, NF-κB, TNF-α, and IL-6 expressions	59
Water extract of *Rhodiola rosea*	PD	Inhibits MAO-A and MAO-B activities and prevents the degradation of important neurotransmitters in PD patients	35
	CVD	Reduces iNOS expression	55
	Pulmonary hypertension	ACE-inhibitory activity	56
	CVD	Causes withdrawal of sympathetic vasomotor tone and the circulatory angiotensin system	60
	STZ-induced diabetes	Increases β-endorphin secretion from adrenal glands to activate opioid μ-receptors	72
	Leukemia	Increases intracellular ROS in K-562 cell line; induces apoptosis, drives the cell to an oxidative-stress-induced cell death; arrests cell-cycle progression at G_2_/M	84
Ethanol extract of *Rhodiola rosea*	Pulmonary hypertension	ACE-inhibitory activity	56
	Diabetes	Inhibits the activities of α-amylase, α-glucosidase, and ACE	56
	Diabetic nephropathy	Lowers the expression of TGF-β1 in renal tissues	71
Polysaccharide from *Rhodiola rosea*	T lymphocytes in tumors	Increases the spleen and thymus indices and the production of cytokines (IL-2, TNF-α, and IFN-γ); increases the CD4^+^/CD8^+^ ratio	86
Tyrosol	Diabetes	Inhibits the activity of α-glucosidase	56
Oligomeric proanthocyanidin (OPCRR)	AD	Increases SOD and GSH-Px activities	19
Methanol extract of *Rhodiola rosea*	PD	Inhibits MAO-A and MAO-B activities and prevents the degradation of important neurotransmitters in PD patients	35
Ethanol extract of *Rhodiola crenulata*	Diabetes	Inhibits α-amylase, α-glucosidase, and ACE activities	56
Water extracts of *Rhodiola crenulata*	Diabetes	Inhibits α-amylase, α-glucosidase, and ACE activities	56
3% rosavin and 0.8% salidroside from *Rhodiola rosea*	Depression	Increases the blood-brain barrier permeability to precursors of DA and 5-HT;induces neural stem cell proliferation in the hippocampus	26
*Rhodiola crenulata* root extract	Hepatoma	Increases glycogen synthesis and the expression of regulatory enzymes in HepG2 cells; suppresses fat accumulation in hepatic cells under high-glucose conditions; is associated with the AMPK signaling pathway	77
*Rhodiola crenulata* root extract	Diabetes	Suppresses fructose-induced hyperinsulinemia and increases the insulin resistance index by modulating sarcolemmal and intracellular CD36 redistribution	69
Water extract of radix et rhizoma *Rhodiola kirilowii*	AMI	Elevates the expressions of HIF-1α, HIF-1β, and VEGF	59
*Rhodiola rosea* extract	CVD	Increases the levels of endogenous opioid peptides	54
Extract of *Rhodiola rosea*	Hypomnesia	Regulates the expression of monoamines and opioid peptides to increase the adaptability and activity of the central nervous system	40
		Modulates the activity and levels of ACh in the brain	38
		Increases the levels of NE, DA, 5-HT and ACh	39
Extract of *Rhodiola rosea*	PD	Facilitates production and proliferation of dopamine-producing cells	36

## Chemical constituents of *Rhodiola*

*Rhodiola* contains salidroside, flavonoids, terpenoids, sterols, tannins and many other compounds. These chemical constituents are the main focus for the investigation of Rhodiola biological activities. Since salidroside is the main bioactive constituent of *Rhodiola* and it is distributed in all parts of the plant and has various biological activities. This review focuses primarily on the application of salidroside and *Rhodiola* extract for the treatment of aging-related diseases.

### Effects of *Rhodiola* on aging-related diseases

#### Effects of salidroside and Rhodiola extract on AD

Alzheimer’s disease is an age-related neurodegenerative disorder, which presents as learning and memory dysfunctions at an early stage and eventually evolves into cognitive disorder. The key risk factor for developing AD is age [1, 2].

The mechanisms of AD may be related to the deposition of β-amyloid (Aβ) peptide and intracellular neurofibrillary tangles consisting of hyperphosphorylated tau protein, which are important characteristics of AD and can lead to serial neuronal loss and brain atrophy [3,4]. Salidroside reduced neurodegeneration in tau-transgenic Drosophila and inhibited neuronal loss by upregulating phosphorylated GSK-3β (p-GSK-3β) and downregulating phosphorylated tau [5]. It also reduced Aβ levels and Aβ deposition in the brain by upregulating phosphatidylinositide 3-kinase (PI3K)/AKT signaling, thus reducing Aβ-induced cognitive impairment in rats [6,7]. In SH-SY5Y cells, salidroside also weakened the hypoxia-induced abnormal processing of amyloid precursor protein (APP), which is another risk factor for AD because abnormal APP generates significant Aβ [8].

Research has shown that oxidative stress plays an important role in the progression of AD [9]. Salidroside protects neurons from oxidative stress by activating antioxidant enzymes thioredoxin (TRX), hemeoxygenase 1 (HO-1), and peroxiredoxin 1 (PRX1). Salidroside also reduces the expression of the proapoptotic protein BAX and increases the level of antiapoptotic protein BCL-XL [8]. Neuronal apoptosis in the hippocampus is related to AD. Salidroside ameliorated the AD-associated cognitive deficit by preventing the activation of caspase 3, increasing the BAX/BCL-2 ratio, and reversing hippocampal neuronal loss caused by chronic cerebral hypoperfusion in rats. The study also showed that salidroside reduced apoptosis in the hippocampal CA1 area [10].

Disturbed energy metabolism is another main cause of AD thus, mitochondrial damage has been the focus of many AD studies. Salidroside greatly reduced cell damage and protected the mitochondria against damage induced by sodium azide [11], indicating that salidroside improves mitochondrial function.

The biotransformation of APP to Aβ is considered a very important step in the deposition of Aβ [3]. Changes in APP metabolism plays a key role in the long-term action of acetylcholinesterase (AChE) inhibitors [3, 12], and the level of acetylcholine (ACh) in AD brain tissues is significantly reduced [13]. *Rhodiola rosea* extracts (50 µg/ml) in different solvents (H2O, EtOAc, or BuOH) reduced AChE by 25.8%, 49.1%, and 40.8%, respectively. At a concentration of 100 µg/ml, extracts in the different solvents conferred 7.6%, 29.3%, and 24.3% neuroprotection, respectively. These findings suggest that the *Rhodiola rosea* extract has potential therapeutic effects for AD, especially the EtOAc-based extract [14].

Recent studies have closely examined antioxidants as therapeutic strategies for patients with AD [15-18]. One active ingredient of *Rhodiola rosea* - oligomeric proanthocyanidin (OPCRR) - has demonstrated significant antioxidant activity. OPCRR significantly increased superoxide dismutase (SOD) and glutathione peroxidase (GSH-Px) activities and reduced malondialdehyde (MDA) content in the serum, heart, liver, and brain tissues of mice, suggesting that OPCRR is a potent natural antioxidant that can be used for the treatment of AD [19]. Therefore, *Rhodiola rosea* may also be a potent antioxidant with potential therapeutic effects in patients with AD.

#### Effects of salidroside and Rhodiola extract on depression

Depression is a common mental health disorder characterized by sadness, loss of interest or pleasure, sense of guilt, feeling of shame, tiredness, disturbed sleep or appetite, and poor concentration. Research has demonstrated that salidroside may have antidepressant activity in mice [20].

Inflammation plays an important role in the development of depression. The serum levels of proinflammatory cytokines such as interleukin 6 (IL-6), tumor necrosis factor α (TNF-α), and IL-1β are elevated in major depressive disorders. A study that evaluated the antidepressant effects of salidroside on olfactory-bulbectomized (OBX) rats showed that salidroside treatment significantly reduced TNF-α and IL-1β levels in the rat hippocampus [21]. Salidroside also significantly attenuated the levels of IL-6 and TNF-α in mice with depression-like behavior. 5-Hydroxytryptamine (5-HT) and norepinephrine (NE) in the brain are known to play important roles in the pathogenesis of depression; treatment with salidroside significantly attenuated NE and 5-HT levels in the prefrontal cortex in mice [22].

Brain-derived neurotrophic factor (BDNF), an important member of the neurotrophic protein family, is important in the pathophysiology of depression. Western blotting analysis showed that BDNF was increased in the rat hippocampus after treatment with salidroside, suggesting that salidroside plays a neuroprotective role by regulating the BDNF/TRKB signaling pathway [23].

The effects of *Rhodiola rosea* extract on depression were documented in a phase III randomized double-blind placebo controlled clinical trial. In the 6-week study, oral administration of *Rhodiola rosea* extract at a dose of 170 mg per day significantly reduced overall depressive symptoms including insomnia, emotional instability, and somatization, relative to those in the placebo-treated group [24].

The effects of an alcohol extract of *Rhodiola rosea* (usually containing 3% rosavin and 1% salidroside) on the central nervous systems of mice has been investigated. The extract displayed significant antidepressant-like activity in mice, which was independent of the dose [25]. A commercial *Rhodiola rosea* product (SHR-5, from Swedish Herbal Institute, Goteborg, Sweden, containing 3% rosavin and 0.8% salidroside) is considered to play a key role in stress modulation and the adaptability of the body. Its mechanism may be related to its ability to increase the permeability of the blood-brain barrier to the precursors of dopamine (DA) and 5-HT [26]. The administration of *Rhodiola rosea* has also been shown to improve concentration and reduce the stress response [27, 28].

A recent study indicated that depression is associated with suppressed cellular proliferation and apoptotic changes in hippocampal tissue [29]. *Rhodiola rosea* extract improved 5-HT levels in the hippocampi of depressive rats, and low doses induced neural stem cell proliferation in their hippocampi, which may have repaired the injured hippocampal neurons [26].

#### Effects of salidroside and Rhodiola extract on other central nervous system diseases

Ischemic cerebrovascular disease is a serious malady often affecting the elderly [30]. In a rat ischemia-reperfusion model of cerebrovascular disease, the antioxidative activity of salidroside significantly attenuated cerebral ischemic injury [31].

Polyglutamine (polyQ) aggregation plays a prominent role in the pathological process of Huntington’s disease. In a transgenic *Caenorhabditis elegans* model, salidroside reduced neuronal death and behavioral dysfunction mediated by polyQ toxicity [32]. Oxidative stress is known to be involved in status epilepticus; in a kainic-acid-induced model of status epilepticus, salidroside showed a neuroprotective effect by regulating AMPK/SIRT1/FOXO1 signaling [33].

PD is a neurodegenerative disease characterized by the progressive loss of dopaminergic neurons in the substantia nigra. Monoamine oxidase (MAO) is an important enzyme that modulates the metabolic recycling of catecholamines and 5-HT in the central nervous system [1, 34]. MAO-A and MAO-B are two important oxidases found at high levels in PD patients. Inhibition of these oxidases is currently used as a treatment for PD. Extracts of *Rhodiola rosea* contain both MAO-A and MAO-B inhibitors, which prevent the degradation of important neurotransmitters in PD patients [35]. *Rhodiola rosea* may also facilitate the production and proliferation of dopamine-producing cells [36].

Hypomnesia is a common symptom in the elderly. Oxidative damage and neuronal injury are two main causes of hypomnesia. In a randomized double-blind placebo-controlled clinical trial, an extract of *Rhodiola rosea* improved and enhanced the accuracy of memory [37]. *Rhodiola* could also alleviate learning and memory impairments in rats with scopolamine-induced memory loss through its neuroprotective capability [38]. Many studies have shown that *Rhodiola rosea* increased the levels of NE, DA, 5-HT and ACh, which stimulated central nervous system activity and improved learning and memory [39]. An extract of *Rhodiola rosea* showed a beneficial effect on learning and memory processes in mice with scopolamine-induced memory impairment possibly through modulation of brain ACh levels. An extract of *Rhodiola* also improved the performance of rats in a water maze task and partly reversed the impairment of learning and memory induced by scopolamine [38]. These activities of *Rhodiola rosea* extract may be related to its regulation of the ACh, monoamines and opioid peptides, thereby increasing the adaptability and activity of the central nervous system [40]. Therefore, *Rhodiola rosea* may effectively improve hypomnesia.

#### Effects of salidroside and Rhodiola extract on CVD

CVD is one of the most common diseases in the world and includes disorders of the heart and/or blood vessels such as coronary artery diseases, heart failure, heart arrhythmia. Although our understanding of CVD has improved, it is still the leading cause of death worldwide, especially in the elderly population [41]. Two mechanisms known to be involved in ischemic heart disease are oxidative stress and cellular apoptosis. Many studies have demonstrated that salidroside protects against myocardial ischemia-reperfusion injury and myocardial hypoxic injury.

“Oxidative stress” refers to the imbalance between the oxidative and antioxidative systems in the body. The oxidative system includes reactive oxygen species (ROS) and reactive nitrogen species, whereas the antioxidative system includes various enzymes, such as SOD and GSH-Px [42]. Salidroside may attenuate hydrogen peroxide (H_2_O_2_)-induced cell damage by downregulating Ca^2+^ and ROS via a cAMP-dependent pathway [43]. In a study using human umbilical vein endothelial cells (HUVECs), pretreatment with salidroside protected the cells from H_2_O_2_-induced injury by promoting mitochondrial biogenesis and its related functions [44, 45]. Using bone-marrow-derived endothelial progenitor cells (BM-EPCs), salidroside reduced the intracellular levels of ROS and increased H_2_O_2_-induced phosphorylation of AKT and ERK1/2 and simultaneously reduced H_2_O_2_-induced phosphorylation of JNK and p38 MAPK. Therefore, salidroside exerts protective effects against oxidative-stress-induced endothelial injury [46].

Antioxidant enzymes including SOD and GSH-Px are biocatalysts produced by living cells that can slow the rate of oxidation. MDA is the end-product of ROS-induced lipid peroxidation in organisms. Creatine kinase (CK) activity is thought to be more reliable than electrocardiography in the diagnosis of myocardial infarction, and CK-MB, an isoenzyme of CK, has the highest diagnostic specificity. Studies of the cardioprotective effects of salidroside showed that salidroside significantly reduced the levels of CK, CK-MB, and lactate dehydrogenase induced by exhaustive swimming. Salidroside also significantly increased GSH-Px and SOD activities and reduced the MDA content in rat liver tissue [47, 48], indicating that salidroside could protect the heart from repeated exhaustive injury.

Apoptotic cell death is an important mechanism of myocardial ischemia-reperfusion injury. Therefore, antiapoptotic strategies could be used to prevent reperfusion injury to the heart. Salidroside has been shown to regulate apoptosis in CVD. For example, a study by Zhang et al. revealed that salidroside significantly increases the levels of vascular endothelial growth factor (VEGF), upregulates the expression of hypoxia inducible factor 1α (HIF-1α) protein and induces its translocation. These data indicate that salidroside exerts a protective effect against hypoxia-induced cardiomyocyte necrosis [49]. Salidroside also reduces the expression of BAX and restores the balance of pro- and anti-apoptotic proteins. It protected endothelial cells from cobalt-chloride-induced apoptosis by regulating the expression of BCL-2 family proteins [50]. Furthermore, salidroside inhibited myocardial apoptosis in a rat model of acute myocardial infarction (AMI) and reduced the ischemia-mediated myocardial damage by increasing the phosphorylation of AKT and reducing the activation of caspase 3, markedly increasing the BCL-2/BAX ratio, and preserving the mitochondrial transmembrane potential [51]. Salidroside also provided protection against coxsackievirus B3, which could cause viral myocarditis, both *in vitro* and *in vivo* [52, 53].

*Rhodiola rosea* extract, at a dose of 3.5 mg/kg given orally, prevented the reperfusion-induced loss of contraction strength in an isolated reperfused rat heart and prevented the reduction of coronary blood flow and the development of contracture in the post ischemic period. This effect is probably related to the increased levels of endogenous opioid peptides induced by *Rhodiola rosea* [54]. The expressions of inducible nitric oxide synthase (iNOS) mRNA and protein increased markedly with myocardial ischemic injury while administration of a water extract of *Rhodiola rosea* downregulated the expression of iNOS in a dose-dependent manner. Therefore, the protective mechanism of *Rhodiola rosea* against ischemic injury to the heart may be through its downregulation of iNOS expression [55].

Pulmonary hypertension (PH) is a chronic, complex, and progressive disease that can lead to death. In general, *Rhodiola* species have an inhibitory effect on the development of hyperglycemia and hypertension. Both ethanol and water extracts of *Rhodiola rosea* showed strong angiotensin-converting enzyme (ACE)-inhibiting activities [56].

*Rhodiola rosea* is beneficial for high-altitude-related symptoms and the acute exacerbation of PH. The attenuation of PH by *Rhodiola rosea* has been demonstrated experimentally in chronically hypoxic rats. These beneficial effects may be related to its possible regulation of signaling via several growth factors including endothelin 1, nitric oxide, VEGF, ACE, nuclear factor κB (NF-κB), TNF-α, and IL-6. Changes in these growth factors have been implicated in the pathogenesis of PH, which manifests as chronic pulmonary vasoconstriction, vasoproliferation, and vascular inflammation [57].

N-Tyrosol has an anti-arrhythmia activity, which could reduce the incidence of ventricular tachycardia and fibrillation, increase the percentage of animals without ventricular arrhythmia, and mitigate the severity of ventricular arrhythmia [58]. The rhizome of *Rhodiola kirilowii* significantly promoted the expression of the von Willebrand factor in the infarct and non-infarct zones of the rat myocardium. *Rhodiola kirilowii* also promoted angiogenesis in the myocardia of rats with AMI by elevating the expressions of HIF-1α, HIF-1β, and VEGF [59].

The systemic administration of the water-soluble fraction of *Rhodiola rosea* sacra radix induced potent hypotensive activity, which was mediated by the withdrawal of sympathetic vasomotor tone and the circulatory angiotensin system. The positive inotropic and chronotropic effects of the water-soluble fraction may be attributable to its direct vagal inhibition in the heart [60].

#### Effects of salidroside and Rhodiola extract on diabetes

With the improvement of living standards, people live longer. This increases the prevalence of diabetes, which is the third most serious threat to human health after CVD and cancer [61]. Traditional Chinese medicine is currently used in the treatment of diabetes and its associated complications. Recent studies have demonstrated that *Rhodiola rosea* extract and salidroside have potent effects on curbing diabetes.

Diabetes increases oxidative stress [62] by increasing the mitochondrial production of the superoxide anion, non-enzymatic glycation of proteins, and glucose autoxidation [63-65]. A previous study observed that salidroside administered daily to mice significantly reduced fasting blood glucose, total cholesterol, triglyceride, and MDA levels, while increasing serum insulin levels and SOD, GPx, and catalase activities. These results indicate that salidroside protects against experimental diabetes, possibly through the reduction of diabetes-induced oxidative stress [66].

Vascular disease is regarded as a severe complication in diabetes mellitus. Hyperglycemia and hypertension are recognized as two of the leading risk factors for vascular complications in diabetic patients. When used in a combination therapy, salidroside simultaneously lowered blood glucose and blood pressure in diabetic rats. The mechanism involved the inhibition of the function and expression of L-type Ca^2+^ (Ca_L_) channels in vascular smooth muscle cells [67]. Almost half of all diabetic patients also suffer from intractable neuropathic pain. Salidroside was reported to ameliorate diabetic neuropathic pain in rats by inhibiting neuroinflammation and the expression of P2X7 receptors [68]. Insulin resistance is also a serious problem in diabetes and the *Rhodiola rosea* extract appears to affect insulin resistance. The *Rhodiola crenulata* extract significantly suppressed fructose-induced hyperinsulinemia and increased the homeostasis model assessment of insulin resistance (HOMAR-IR) index and the adipose tissue insulin resistance index in male Sprague Dawley rats, indicating that the amelioration of insulin resistance by *R. crenulata* extract is attributable to its modulation of the sarcolemmal and intracellular redistribution of CD36 [69].

When an ethanol extract of *Rhodiola rosea* was administered orally to streptozocin (STZ)-induced diabetic rats, the extract showed a significant anti-hyperalgesic effect [70]. Another study revealed that an ethanol extract of *Rhodiola rosea* protected diabetic rats from early nephropathy, which may have been associated with the reduced the expression of transforming growth factor β1 (TGF-β1) in renal tissues [71]. A water extract of *Rhodiola rosea* administered orally to STZ-diabetic rats lowered plasma glucose and improved hyperglycemia, acting via an increase in β-endorphin secretion from the adrenal gland, which activated the opioid μ-receptors [72].

In another study, *R. crenulata* and *Rhodiola rosea* exerted significant inhibitory activities on α-amylase, α-glucosidase, and ACE. Furthermore, a major phenolic component of *Rhodiola* species, tyrosol, has a strong α-glucosidase-inhibitory activity, and may have utility as an effective therapy for postprandial hyperglycemia in patients with type II diabetes [56].

Taken together, *Rhodiola* species are potential pharmaceutical agents for the treatment of diabetes mellitus.

#### Effects of salidroside and Rhodiola extract on liver disease

Hepatic fibrosis is a common pathological basis for chronic liver disease and cirrhosis. Recent studies have demonstrated that the activation and proliferation of hepatic stellate cells (HSCs) are key factors in hepatic fibrogenesis, in which oxygen free radicals and lipid peroxidation play important roles. Salidroside has antioxidant activity, which significantly reduces the production of intracellular MDA and GSH, increases SOD and GSH-Px activities, markedly inhibits the proliferation of HSCs, and significantly lowers the levels of collagen *in vitro*. The mechanism of salidroside action on fibrosis may be related to its inhibition of lipid peroxidation [73].

In a mouse model of acute liver fibrosis induced with D-galactosamine and lipopolysaccharide, salidroside reduced elevated serum levels of aspartate amino transferase and alanine amino transferase as well as serum TNF-α and NO in a dose-dependent manner. It restored the depleted hepatic GSH, SOD, catalase and GSH-Px activities, and reduced MDA levels in the liver. Salidroside also reduced the degree of necrosis, caspase 3 and HIF-1α levels in the mouse liver. The hepatoprotective mechanism of salidroside may be related to its antioxidant activity and the functional inhibition of HIF-1α [74].

Oxidative stress is implicated in the functional impairment of adipose tissue and other tissues such as the liver. A *Rhodiola* extract protected the liver from oxidative-stress-induced damage while a methanolic extract of *R. sachalinensis* protected against D-galactosamine-induced cytotoxicity in primary mouse hepatocyte culture [75].

The liver is an important organ in the homeostasis of body energy because it regulates glucose and lipid metabolism, which are linked to the pathological progression of cirrhosis, non-alcoholic fatty liver disease, hepatitis, and liver cancer [76]. Both *in vitro* and *in vivo* experiments have demonstrated that *R. crenulata* root extract increased glycogen synthesis and the expression of regulatory enzymes in HepG2 cells and suppressed fat accumulation in hepatic cells under high-glucose conditions. These results indicate that the regulatory effects of *R. crenulata* root extract on hepatic glycogen and lipid metabolism are associated with the AMPK signaling pathway [77].

#### Effects of salidroside and Rhodiola extract on cancer

The incidence of cancer increases markedly with age. People over the age of 65 years are generally more susceptible to cancer. The mechanism underlying this phenomenon may involve the accumulation of mutations in critical genes, which alter the normal programs of cell proliferation, differentiation, and death [78].

Salidroside selectively inhibited the growth of bladder cancer cell lines and TP53-defective cells, whereas it had little effect on non-malignant bladder epithelial cells. This may be attributable to its inhibition of the mTOR pathway and its induction of autophagy [79].

Oxidative stress is an important apoptotic stimulus in cancer cells and plays a critical role in both tumorigenesis and metastasis. Salidroside has potent antioxidant properties and could significantly inhibit the proliferation of A549 cells by inducing G0/G1 phase arrest and apoptosis. These activities could be related to its reduction of intracellular ROS generation and phosphor-p38 MAPK expression in A549 cells [80].

Another *in vitro* study demonstrated the inhibitory effects of salidroside on tumor cell metastasis in human fibrosarcoma HT1080 cells, mediated by the downregulation of the ROS/PKC/ERK1/2 signaling pathway [81]. Salidroside also inhibited the migration and invasion on colon carcinoma SW1116 cells via the JAK2/STAT3-dependent pathway [82], and significantly reduced tumor-induced angiogenesis in mice [83].

An aqueous extract of the *R. imbricata* rhizome also reduces the proliferation of K-562 cells, increased intracellular ROS in the K-562 cell line, induced apoptosis and cell death. This extract also arrested cell-cycle progression at G2/M. These observations suggest that an aqueous extract of the *R. imbricata* rhizome has potent anticancer activities and might be useful in the treatment of leukemia [84].

The immune system plays an important role in the regulation and prevention of cancer [85]. A polysaccharide from *Rhodiola rosea* is a promising candidate for reducing immune suppression in experimental tumors. In an *in vitro* experiment, the polysaccharide had an acute cytotoxic effect on Sarcoma 180 (S-180) cells. In an *in vivo* experiment, it inhibited the growth of S-180 cells, dramatically enhanced immune responses, and protected the vital internal organs of S-180-bearing mice. These activities were associated with increases in the spleen and thymus indices, the production of cytokines (IL-2, TNF-α, and interferon-γ), and the CD4^+^/CD8^+^ ratio in the tumor-bearing mice. These findings indicate that this polysaccharide from *Rhodiola rosea* is a novel and promising immune therapeutic agent for the treatment of cancer [86].

### Conclusions

Traditional Chinese medicine offers a different perspective for the treatment of ailments and its role in anti-aging is garnering increasing attention [87-91]. *Rhodiola* is one of the most important and frequently used traditional Chinese herbal medicines. Among the chemical components of *Rhodiola* such as salidroside, polysaccharides, flavonoids, terpenoids, and many others, salidroside is the main bioactive agent. Many extracts and single compounds isolated from *Rhodiola* have shown pharmacological effects on different organs and systems, including the cardiovascular and nervous systems. *Rhodiola* extracts and compounds display numerous bioactivities including anti-oxidative, anti-aging, anti-cancer and neuroprotective activities as well as possess significant adaptogenic properties.

Although *Rhodiola* is commonly used with other herbs in traditional Chinese medicine, many studies have indicated that this medicinal herb exerts significant pharmacological effects on its own. The pharmacology of some traditional uses of *Rhodiola* has been validated in recent studies, although these studies were primarily conducted *in vitro*. Therefore, the effects of these compounds require verification *in vivo*. Approximately 200 chemical compounds have been isolated from *Rhodiola* species, but only a few compounds have been investigated including salidroside, flavonoids, and polysaccharides. The safety of these components in clinical practice must also be addressed. Unfortunately, there are only a few toxicological studies of these extracts and compounds, while *in vitro* and *in vivo* may not be applicable to humans. To explore the full medicinal potential of *Rhodiola*, the gaps between experimental results and clinical applications must be bridged by new innovations.

In conclusion, the evidence presented in this review strongly supports the proposition that *Rhodiola* has therapeutic properties for a variety of age-related diseases and therefore warrants further investigation.
